# The protective effects of silymarin nanoemulsion on 5-fluorouracil-induced gastrointestinal toxicity in rats

**DOI:** 10.1016/j.jsps.2023.06.005

**Published:** 2023-06-16

**Authors:** Soheila Safarpour, Samaneh Safarpour, Ali Akbar Moghadamnia, Sohrab Kazemi, Anahita Ebrahimpour, Fatemeh Shirafkan

**Affiliations:** a Student Research Committee, Babol University of Medical Sciences, Babol, Iran; b Department of Pharmacology and Toxicology, School of Medicine, Babol University of Medical Sciences, Babol, Iran; c Department of Oncology, Faculty of Medicine and Dentistry, University of Alberta, Edmonton, Canada; d Cellular and Molecular Biology Research Center, Health Research Institute, Babol University of Medical Sciences, Babol, Iran

**Keywords:** Silymarin, Gastrointestinal toxicity, Oxidative stress, IL-2, TNF-α, 5-Fluorouracil

## Abstract

5-Fluorouracil (5FUra) is the third most popular chemotherapeutic component employed to treat solid tumors. In the present study, we aimed to appraise the silymarin (SM) and silymarin nanoemulsion (SMN) effect on 5FUra-induced gastrointestinal toxicity in adult male rats. A total of 30 male Wistar rats were divided into 6 groups including the control (Crl) group, and groups treated with SMN (5 mg.kg^−1^), SM (5 mg.kg^−1^), 5FUra + SMN (5 mg.kg^−1^), and 5FUra + SM (5 mg.kg^−1^) by IP injection for 14 days. And gastrointestinal toxicity was induced by a single intraperitoneal (IP) injection of 5FUra (100 mg.kg^−1^) for the last group in the study. Treating rats with SM and SMN diminished elevating malondialdehyde (MDA) levels, and improved total antioxidant capacity (TAC) levels. Also, the intensity of mRNA expression of interleukin-2 (IL-2) and tumor necrosis factor-alpha (TNF-α) caused by 5FUra in the gastrointestinal tissue tract, and macroscopic oral ulcerations decreased, ass well as weight loss was prevented, particularly in the SMN group. Moreover, in the microscopic scope, there were significant improvements in the levels of hyperemia, hyaline, and inflammatory cell infiltration in the tongue, esophagus, and intestinal tissues in the FUra + SMN and FUra + SM groups compared to 5FUra. Hence, treatment with SM and SMN reduced oxidative stress, histopathological degeneration, and gene expression of inflammatory markers in the gastrointestinal tract. According to the results, treatment with SM and SMN markedly decreases the gastrointestinal toxicity caused by 5FUra.

## Graphical abstract

**Figure:**
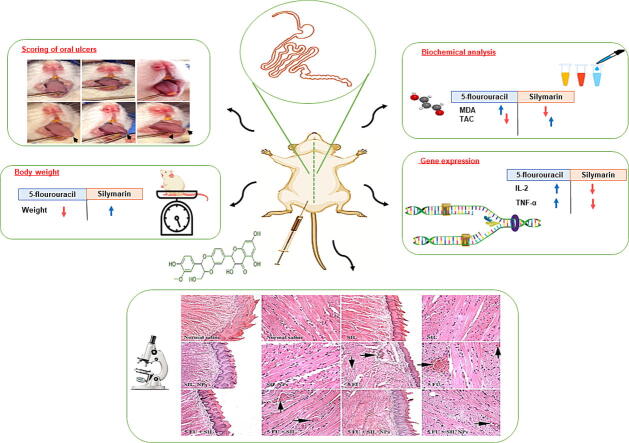

## 1 Introduction

The pyrimidine 5-fluorouracil (5FUra), the principal component and the most popular chemotherapeutic agent, is widely employed in the treatment of a variety of tumors, particularly malignant gastrointestinal tumors, skin, and breast cancers (Anand, 1994, Diasio and Harris, 1989). Anticancer efficacy of 5FUra is imposed via thymidylate synthase suppression and embedment of its metabolites into RNA (Longley et al., 2003, Wigmore et al., 2010). Treating with 5FUra raises fractioning ribosome-free L23, L5, and *L*11 protein levels and their binding to oncoprotein mouse double minute 2 homolog (MDM2), which in turn can activate protein 53 (p53) and arrest the G1/S phase (Sun, Dai, & Lu, 2007).

Despite the 5FUra merits, the dangers of this drug should also be contemplated, since treating with fluoropyrimidine could evoke a spectrum of adverse impacts including myelosuppression, nausea, emesis, mucositis, and toxicity to other organs (Steger, Hautmann, & Kolbl, 2012). Disruptions in the integrity and/or function of the mucosal lining of the gastrointestinal tract are particularly a vital issue in patients receiving chemotherapeutic agents. Mucositis reflecting a self-limited, short-term negative impact of treatment can influence the whole alimentary tract. The spectrum of symptoms includes dysphagia, odynophagia, oral ulcerations, esophagitis, gastritis, malabsorption, and diarrhea (Negrin, 2022).

Natural components isolated from plants have almost been discovered to have efficacious preventive and protective impacts against some pathological states. Silymarin (SM) is a mixture of flavolignans (i.e. silybin, silydianin and silychristin) extracted from *milk thistle Silybum marianum* seeds. Among the flavolignans, silybin or silybinin is the most abundant biologically active compound of silymarin (Zaulet et al., 2017). The SM's ability to preserve against oxidative stress-induced injury, including lipid peroxidation of the membrane and subsequent degradation, is correlated with inhibiting free radicals and reactive oxygen species (ROS) (De La Puerta et al., 1996, Dehmlow et al., 1996), as well as increasing the antioxidant defense (Pascual, Armesto, & Muriel, 1993). Treatment with SM can decrease cytotoxicity and gastrointestinal mucositis intensity (Sasu et al., 2015). Besides, SM has also been demonstrated to exert anti-proliferative, anti-apoptotic, anti-fibrotic, immunomodulatory, and antiviral impacts (Sasu et al., 2015, Tsai et al., 2008) and has been indicated to hamper tumor necrosis factor-alpha (TNF-α) expression (Ahmad et al., 2013).

Despite all of the benefits, clinical application and therapeutic efficiency of SM flavolignans are limited due to their poor bioavailability. The latter is specifically owing to the crystalline state and low water solubility of silymarin flavonolignans at room temperature, and to their poor permanent absorption (Gažák et al., 2004). These limitations have been tackled by developing pharmaceutical preparations including lipid-based delivery systems with raised silybin bioavailability (Javed et al., 2011, Parveen et al., 2011). The lipid medium seems mainly critical, as the chemical and physical features of the lipid carrier broadly influence the solubility of the compound to be delivered.

So here, we examined the protective role of SM and silymarin nanoemulsion (SMN) in 5FUra-induced gastrointestinal mucositis in adult male rats by assessing the occurrence, severity (graded from 0 to 3), and repeated inspections of localization of mucositis caused by 5FUra in the oral cavity of rats and as a part of their treatment. Also, we evaluated changes in body weight, biochemical parameters, and molecular markers of *interleukin-2* (IL-2) and TNF-α in the tongue, esophagus, and intestine as well as histopathological changes in tongue, esophageal and intestinal tissues.

## 2 Materials and methods

### 2.1 Drugs and chemicals

5FUra and SM were provided by Sigma, St. Louis, MO (USA), and Merck Company (Germany), respectively. Polyoxyl 40 hydrogenated castor oil, glyceryl monooleate (GMO) (Wigmore et al.), and polyethylene glycol 400 (PEG-400) were purchased from Sigma Aldrich. Total Antioxidant Capacity (TAC) and Malondialdehyde (MDA) kits were prepared by Teb Pajohan Razi Company, Iran (TPR-MDA-96 T). Tween 80 was obtained from Kavosh Gostar Daru, Iran. The cDNA synthesis and RNA extraction kits (RT-520 and VT-4050) were obtained from Yekta Tajhiz Azma and Sinacolon companies, Tehran, Iran, respectively.

### 2.2 Preparing SM and silymarin-loaded nanoemulsion (SMN)

To produce a spontaneously formed SMN in an oil phase of PEG 400, polyoxyl 40 hydrogenated castor oil, and glycerylmonooleate (GMO) (1:8:1), 50 mg of SM was added to 10 g of the oil phase. SM, surfactant, cosurfactant, and oil were stirred for 2 h at 100 rpm. More sonication was performed for 1 h using a bath sonicator (Elmasonic Med 60) to complete the mixing process. Then, deionized water was added to the oil phase at a ratio of 5:1 and stirred gently to obtain a nanoemulsion (Safarpour et al., 2022a, Safarpour et al., 2022b).

### 2.3 Zeta potential, particle size, and loading content

The surface charge of nanoemulsions (zeta potential), size distribution, polydispersity index (PDI), and mean size of the SMN were assessed using the dynamic light scattering (DLS) technique by Nano-ZS ZEN 3600 (Malvern Instruments Ltd., England). And the scattering intensity was evaluated at 25 °C and an angle of 90° (Rashedi et al., 2019, Xu et al., 2007).

Loading content (LC%) was measured by the following Eq (Ilk, Sağlam, Özgen, & Korkusuz, 2017):

LC (%) = (Weight of SM in nanoemulsions / Weight of nanoemulsions) × 100.

### 2.4 Observation of nanoemulsion morphology

Scanning Electron Microscopy (SEM) (Quanta FEG 250; FEI, North America) was employed for the surface morphology of the nanoemulsions (Ilk et al., 2017).

### 2.5 HPLC analysis of SM

Briefly, SM was assessed by HPLC supplied with a reverse-phase C18 column (pore size 5 μm, 150 mm × 4.6 mm, ProntoSIL), a vacuum degasser controlled by Smartline-2600 software, and a UV detector. Methanol and water (70:30 v/v) (pH modified to 3.64 with glacial acetic acid) were employed as the mobile phase. To separate SM, an isocratic condition with a temperature of 25 °C in column, 20 μL injection volume, constant flow rate of 1.0 mL/min, and a detection wavelength of 275 nm, was prepared. The calibration curves were created to detect the linear association between the concentrations and the peak areas. The coefficient of determination (r2), intercept, and slope was measured as regression parameters by weighted linear regression (Najafabadi, Kazemipour, Esmaeili, Beheshti, & Nazifi, 2018).

### 2.6 Animals and treatment

Thirty adult male Wistar rats (180 ± 20 g) were housed in wire-bottomed individual cages (approval No: IR.MUBABOL.REC.1400.213(at a controlled and standard temperature (of 23 ± 2 °C) and humidity (60 % ± 5 %), and in a 12/12 h light/dark cycle, with food and water available ad libitum based on the protocols of the Research Council of Babol University of Medical Sciences.

Intraperitoneal (IP) injections of animals were conducted (Razavi & Karimi, 2016). The rats were randomly assigned to 6 groups:

1. Control (Crl) group (n = 5): IP administration of normal saline for 14 days.
2. The 5FUra group (n = 5): a single IP administration of 5FUra (100 mg.kg^−1^) on the first day (Mohamed and Safwat, 2016, Sengul et al., 2021).
3. 5FUra + SMN group: a single IP administration of 5FUra (100 mg.kg^−1^) on the first day and then SMN (5 mg.kg^−1^) for 14 days (n = 5).
4. SMN group: an IP administration of SMN for 14 days (5 mg.kg^−1^) (n = 5).
5. 5FUra + SM group: a single IP administration of 5FUra (100 mg.kg^−1^) on the first day and then SM (5 mg.kg^−1^) for 14 days (n = 5) (Razavi & Karimi, 2016).
6. SM group: an IP administration of SM (5 mg.kg^−1^) for 14 days (n = 5).

### 2.7 Macroscopic histopathological analysis

All rats were placed in the animal house on the first day without any treatment to adapt to their environment. For macroscopic analysis, between the fourth and the ninth days of the injection, the damage severity in a totally blind manner, erosion, vasodilatation, erythema, and oral epithelial ulcerations were graded based on the following grading system (de Araujo et al., 2015):

Score 0: completely healthy (without injury) with no vasodilatation or erosion in the surface area.
Score 1: the presence of erythema, despite no surface erosion.
Score 1.5: the presence of vasodilation, existing surface erosion, and severe erythema.
Score 2: the presence of focal ulcers in one or more mucosal faces, but not surpassing 25 % of the surface area, vasodilatation, and severe erythema.
Score 2.5: the presence of cumulative ulcers at around half of the surface area.
Score 3: the presence of accumulative ulcers at around 75 % of the surface area.

### 2.8 The body weight mensuration

The rats in all groups were weighed on the 1st, 5th, 9th, and 13th days of the treatment period to compare weight changes between these days.

### 2.9 The preparation of serum and tongue, esophageal, and intestinal tissues

After performing different treatments, the experimental animals were anesthetized with ketamine/xylazine (IP) at a dose of 75/25 mg.kg^−1^, and then 2 ccs of blood were obtained from the heart to assess biochemical parameters: MDA and TAC. After weighing, the tongue, esophageal, and intestinal tissues were removed through the incision for histopathological staining.

### 2.10 Thiobarbituric acid reactive substance (TBARS) assay

Shortly, following animal anesthetizing and blood sampling, all the serum samples were stored at −80 °C until measuring the biochemical agents. The TBARS was carried out to assess the levels of oxidative stress in the sera. To measure TBARS levels, after adding 1 mL of serum to 2 mL of TBARS reagent, they were heated at 100 °C for 60 mins. Thereafter, samples were placed in the ice bath for 10 mins and a 10 mins centrifuging was performed at 2500 rpm. Then, thiobarbituric acid and MDA will bind with each other to produce a red component. Elisa reader at 535 nm was used to assess the light absorbance (Katalinić et al., 2007).

### 2.11 Ferric reducing antioxidant power (FRAP) assay

The FRAP assay was employed to evaluate antioxidant power in serum samples. After animal anesthetizing, 1.5 mL of prepared FRAP was added to tubes included with sera, and incubation was performed at 5 °C for 37 mins. After incubation, 51 μL of the sample was added to the tubes and blended well. After re-incubation of mixtures for 15 mins at 37 °C, the antioxidant power of the samples was measured by reducing the ferric ion (Fe3 + ) to the ferro one (Fe2 + ). Thereafter, the blue color intensity was evaluated by an Elisa reader at 593 nm (Szőllősi & Varga, 2002)**.**

### 2.12 Histopathological assessment

After anesthetizing animals with ketamine/xylazine (at a dose of 75/25 mg.kg^−1^, IP) and removing their tongue, esophageal, and intestinal tissues, they were prepared for histological and molecular studies. The tongue, esophageal, and intestinal tissue samples were immediately gathered. After being inflated with mild PBS, they were fixed in 4 % paraformaldehyde for 24 h, placed in paraffin, and then sections (5 µm) were provided by microtome (model Leitz 1512, Germany). A mean of 3 fields was taken into consideration for each slide.

To evaluate the histopathological degeneration in the above-mentioned tissues, H & E staining was used. Three animals per group were available, then 7 slides per animal were provided, 3 fields were chosen for each slide, and the inflammation level was calculated. Histological analysis was conducted using Image J software.

Briefly, tissue specimens of the samples were placed in 100 % alcohol for 5 mins, then placed in 96 % alcohol for 5 mins, stained with hematoxylin for another 5 mins, rinsed with running water for 5 mins as well, and stained with eosin for 15 s, immersed in distilled water for decolorization, placed at ethanol 70 % for 15 s, ethanol 95 % for 30 s, absolute ethanol for 1 min, and xylene for 5 mins and eventually pasted with entellan. Evaluation of histology, inflammation of cells, and morphological deformation in the tongue, esophageal, and intestinal tissues were conducted using H&E staining. These tissues were assessed under a microscope (Olympus BX61VS, Japan), then, the results were analyzed using Image J software.

### 2.13 RNA extraction and real-time polymerase chain reaction (RT- PCR)

To assess TNF-α and IL-2 mRNA expression in the tongue, esophagus, and intestinal tissues, the total RNA was immediately isolated based on the instructions of the manufacturer (Pars Tous, Mashhad, Iran), then the extracted RNAs were transferred to −20 °C overnight and then to −80 °C until the cDNA synthesis. Synthesizing of cDNA was performed based on the instructions of the manufacturer (Pars Tous, Mashhad, Iran). qRT-PCR was performed by an ABI Step One Plus RT- PCR System (Applied Biosystem, USA) with the primer sets for TNF-α and IL-2 as target genes and a housekeeping gene (GAPDH). An amount of 10 μL of RT- PCR reaction mixture contains cDNA (1 μL), SYBR-Green (6.25 μL) (Amplicon high Rox master mix, Denmark), nuclease-free water (2.25 μL), and 0.25 μL of 10 pmol of each primer (Robin Teb Gostar, Tehran, Iran). Based on kit instructions, the steps of reverse transcription were conducted at 25 °C for 10 mins, then 37 °C for 60 mins, and then 85 °C for 5 mins. The PCR was conducted by keeping the temperature at 95 °C for 15 mins, afterward, at 95 °C for 40 cycles of 15 s, at 62 °C for 30 s, and then at 72 °C for 30 s followed by melting curve temperature steps. The TNF-α and IL-2 primers are illustrated in Table 4.

### 2.14 Statistical analysis

GraphPad Prism software version 8 was employed to analyze all data. The parametric variables were represented as mean ± SEM. Analysis was conducted using one-way analysis of variance (ANOVA) and two-way ANOVA and a Tukey post-test. Two-way ANOVA was employed for histopathological scoring changes between the groups. The statistical significance criterion was a P-value of less than 0.05.

## 3 Results

### 3.1 Particle size loading and analysis and nanoemulsion morphology

**Fig. 1:**
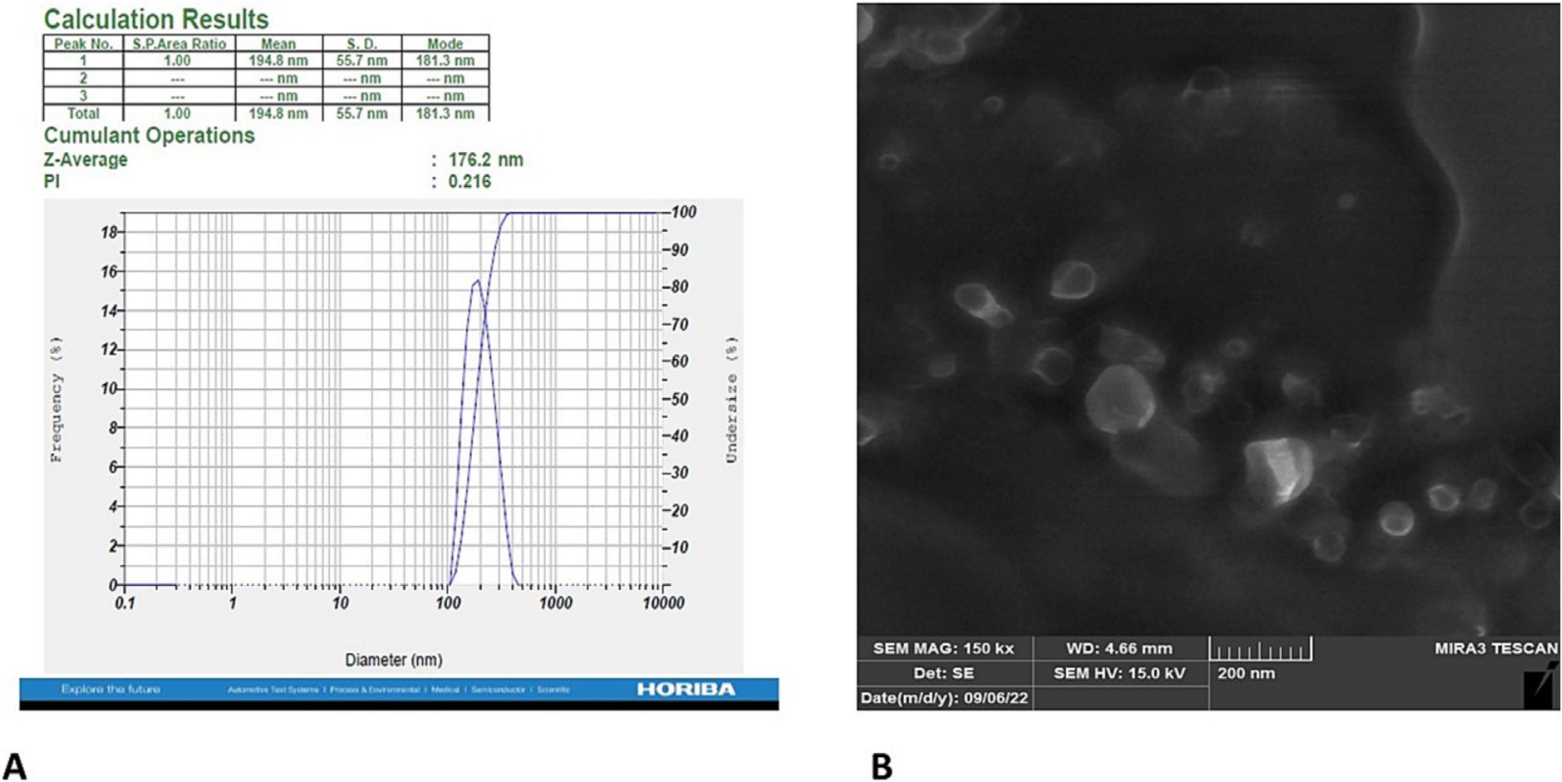
A. Average size and zeta potential of nanoemulsions determined by DLS. B. Scanning electron microscopy images. Morphology and dispersivity of nanoemulsions.

We primarily examined the average size by the DLS method. Fig. 1A has been shown that the average size is 176.2 nm. The size, shape, and surface texture of nanoemulsions were detected using SEM. The high-resolution SEM micrograph of nanoemulsions has been illustrated in Fig. 1B.

The calibration curve was provided by analyzing various concentrations of SMN in the peak area. The concentration of SM was measured by a regression equation created by a calibration curve. The analysis of HPLC indicates that the SMN loading was 68.08 %.

### 3.2 Effect of SM and SMN on oral injury

**Fig. 2:**
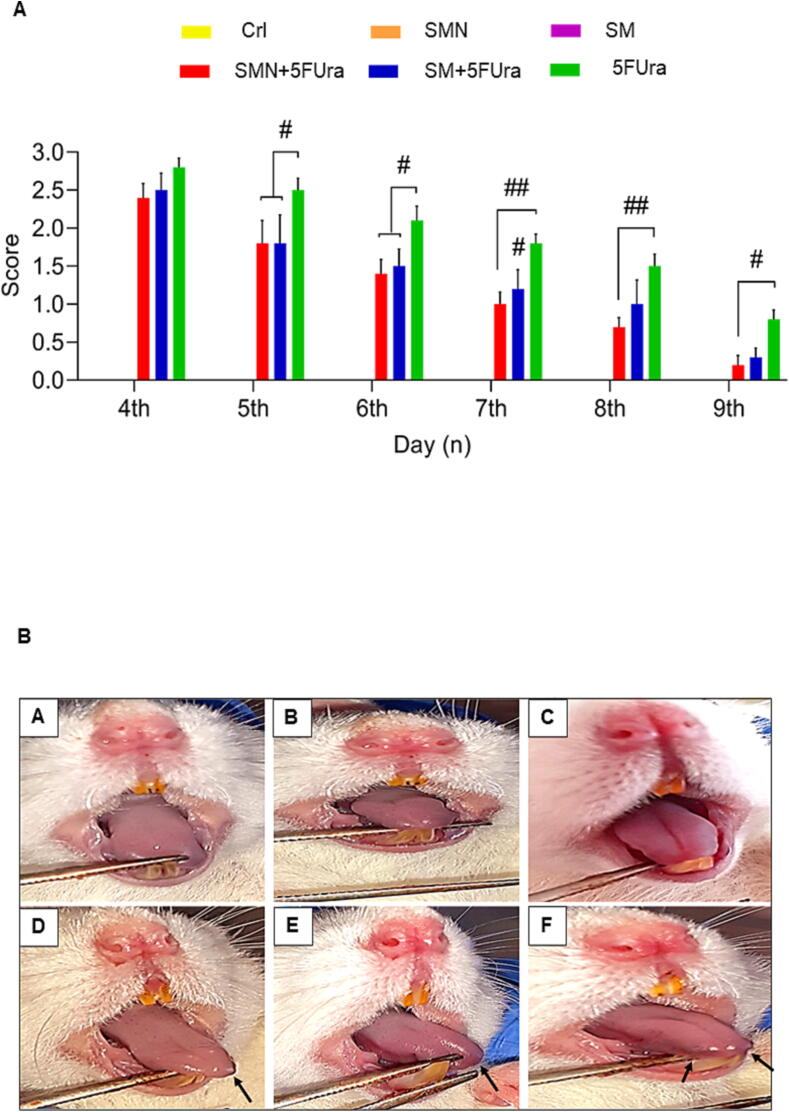
A: Oral injury score in all groups from the 4th day until the 9th day. Data are represented as mean ± SEM. ^#^P-value of less than 0.05, ^##^P-value of less than 0.01: significant in comparison to the 5FUra group. (n = 5) Fig. 2. B: macroscopic analysis of the mouth showed normal and healthy tissues of the tongue and mouth in the Crl (A), SMN (B), and SM (C) groups, and oral ulcers with high intensity in the 5FUra group (F), and lower intensity after treating with SMN + 5FUra (D) and SM + 5FUra (E).

Our study's macroscopic analysis of oral injury showed that healing occurred in oral wounds from days 5 to 9 in groups treated with 5FUra after receiving SM and SMN (Fig. 2. B). Significant reduction of oral ulcers was found during the fifth, sixth, and seventh days in the SM + 5FUra and SMN + 5FUra groups compared to the fifth in the 5FUra group. In addition, on the eighth, and ninth days, substantial improvement of oral wounds was seen only in the SMN + 5FUra group in comparison to the 5FUra group as shown in Fig. 2. A.

### 3.3 Effect of SM and SMN on body weight variation

**Fig. 3:**
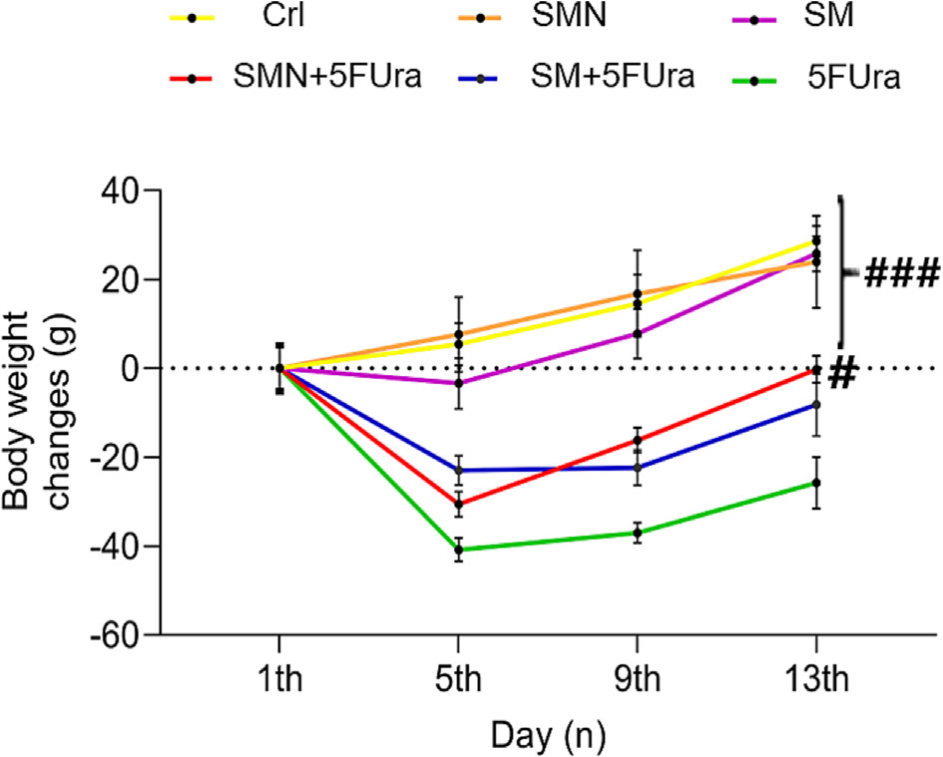
Alterations in body weight of rats in all groups on the first, 5th, 9th, and 13th days of injection. ^#^P-value of less than 0.05: significant in comparison to the 5FUra group. Results are represented as mean ± SEM (n = 5).

Body weight was evaluated on the 1st, 5th, 9th, and 13th days of the study, and body weight alterations were assessed and demonstrated that 5FUra inhibited weight gain compared with the SMN, SM, and Crl groups in the experimental groups indicating growth retardation. There was a substantial difference between the SM and SMN and the 5FUra groups which was particularly seen on the 13th day of the study in rats receiving SMN + 5FUra (Fig. 3). Although SMN and SM prevented the weight loss in the 5FUra group to some extent.

### 3.4 Biochemical analysis

**Fig. 4:**
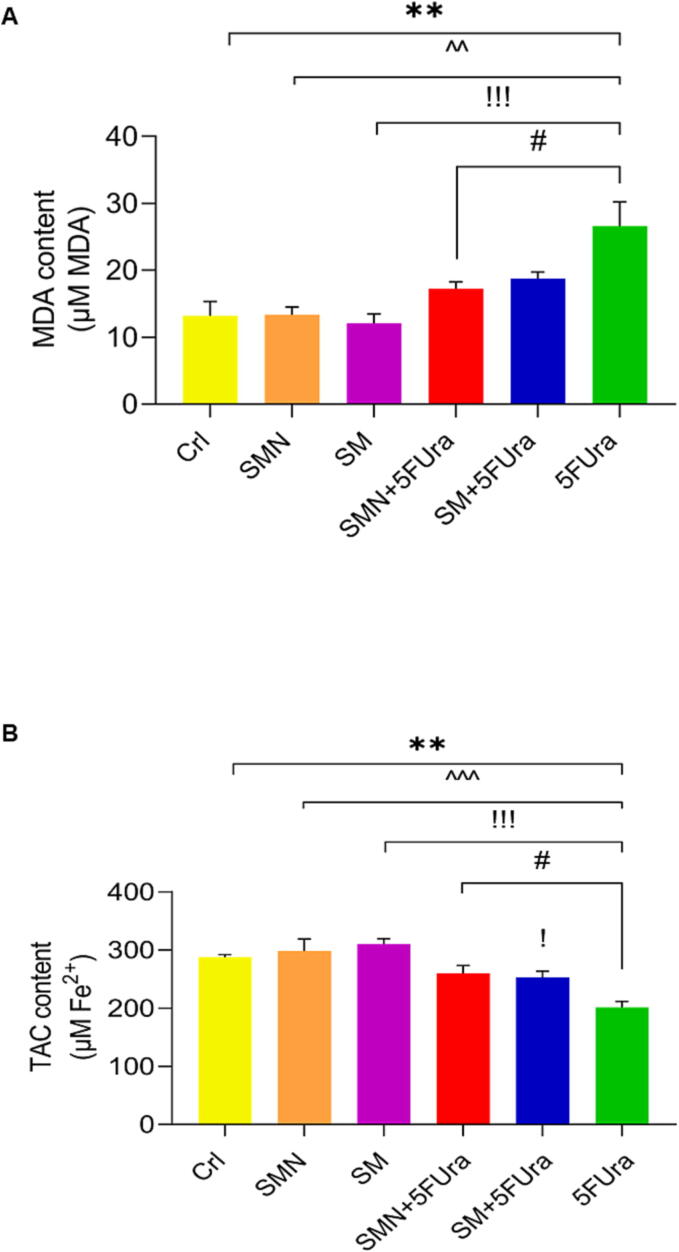
The effect of SM and SMN on biochemical parameters in different groups. (A): MDA, (B): TAC. *P-value of less than 0.05, ^**^P-value of less than 0.01, ^***^P-value of less than 0.001: significant in comparison to the Crl group. ^^^^P-value of less than 0.01 and ^^^^^P-value of less than 0.001: significant in comparison to the SMN group.^!^P-value of less than 0.05,^!!!^P-value of less than 0.001: significant in comparison to the SM group. ^#^P-value of less than 0.05: significant in comparison to the 5FUra group. Results are represented as mean ± SEM (n = 5).

The MDA level was markedly greater in the 5FUra group than that in the SMN, SM, and Crl groups. The levels of MDA in the SM + 5FUra group had a reduction, but this decrease was only substantial in the SMN + 5FUra group in comparison to the 5FUra group (Fig. 4A).

Administrating 5FUra substantially reduced TAC values compared to the SMN, SM, and Crl groups. We showed a markable decrease in TAC values in the SM + 5FUra group compared to the SM group. Furthermore, in SMN + 5FUra and SM + 5FUra groups, growth in the TAC level compared with the rats treated with 5FUra was statistically marked (Fig. 4. B).

### 3.5 Effects of SM and SMN on histopathological alterations of the tongue, esophageal, and intestinal tissues

**Fig. 5:**
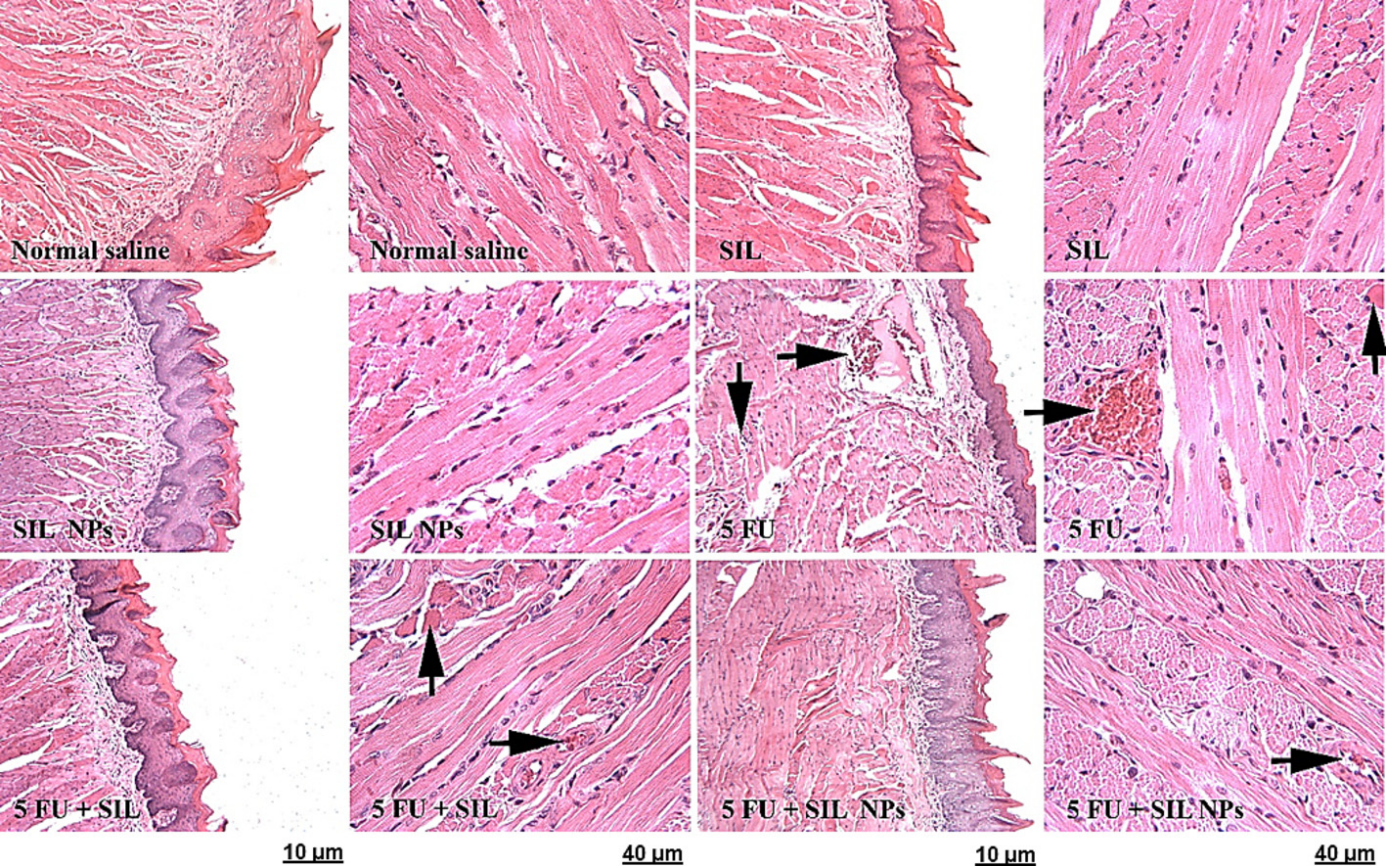

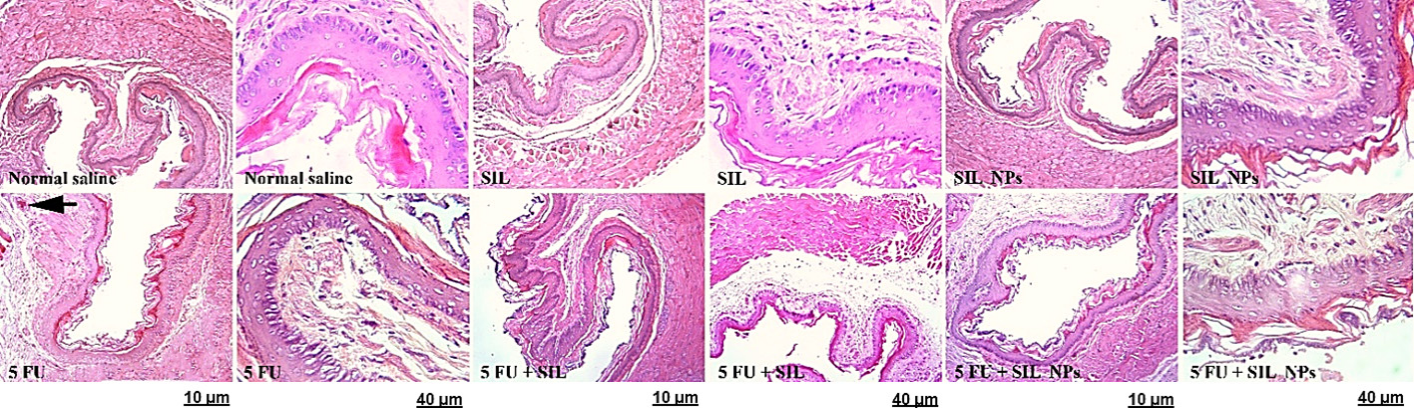

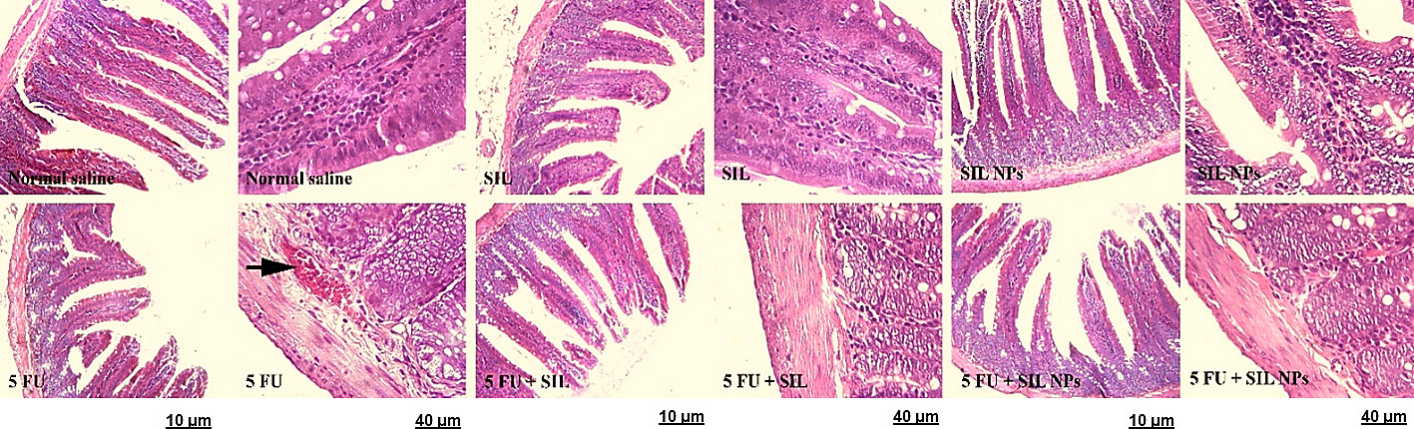
Histopathological alterations of tongue, esophageal, and intestinal tissues using H&E staining. Crl, SMN, and SM groups: normal tissue conditions. Other groups: hyperemia (left arrow), inflammatory cell infiltration (down arrow), and hyaline (up arrow). × 10 magnification, ×40, H&E staining.A Tongue texture. Normal tissue conditions in the Crl, SMN, and SM groups. hyperemia (arrow to the right), inflammatory cell infiltration (down arrow), and hyaline (up arrow) in other groups. B Esophageal tissue. Normal tissue conditions in the Crl, SMN, and SM groups and treatment groups, hyperemia (left arrow) in the 5FU receiving group. C Intestinal tissue. Normal tissue conditions in the groups receiving SMN and SM and treatment groups, hyperemia (arrow to the right) in the 5FUra receiving group.

We evaluated the effect of SM and SMN on histopathological alterations in the tongue, esophagus, and intestine using H & E staining. Results demonstrated a normal cell morphology in the Crl, SMN, and SM groups without any hyperemia and necrosis. Meanwhile, the 5FUra group, demonstrated noticeable levels of tissue histopathological abnormalities and intoxication, for instance, hyperemia, hyaline, and inflammatory cell infiltration in the tongue, esophagus, and intestinal tissues (Table 2, Table 3, Table 4). Also, the groups treated with SMN + 5FUra and SM + 5FUra indicated mild hyperemia in the tongue tissue. In addition, some degree of hyaline in the tongue tissue was seen in the SM + 5FUra group (Fig. 5. A, Table 2). However, rats in the SMN + 5FUra and SM + 5FUra groups, particularly in the SMN + 5FUra group, indicated less tissue injury than in the 5FUra group, demonstrating amelioration of tongue tissue damage (Fig. 5. A, Table 1) and abnormalities in esophageal (Fig. 5. B, Table 2), and intestinal tissues (Fig. 5. C, Table 3) by SMN and SM.

**Table 1:** Histopathological changes with H&E staining in tongue tissue.

Groups	Hyaline	Inflammatory cell infiltration	Hyperemia
Control	**–**	**–**	**–**
SMN	**–**	**–**	**–**
SM	**–**	**–**	**–**
SMN + 5FUra	**–**	**–**	**+**
SM + 5FUra	**+**	**–**	**+**
5FUra	**+**	**+**	**++**

**Table 2:** Histopathological changes with H&E staining in esophageal tissue.

Groups	Hyperemia
Crl	**–**
SMN	**–**
SM	**–**
SMN + 5FUra	**–**
SM + 5FUra	**–**
5Fura	**+**

**Table 3:** Histopathological changes with H&E staining in intestinal tissue.

Groups	Hyperemia
Crl	**–**
SMN	**–**
SM	**–**
SMN + 5Fura	**–**
SM + 5Fura	**–**
5Fura	**+**

**Table 4:** Sequences of primers for IL-2, TNF-α, and the housekeeping genes.

Primer	5′----3′
IL-2 FW	AGAACTCAAACCTCTGGAGGAAG
IL-2 RV	GCTGTCTCATCAGCATATTCACAC
TNF-α FW	AAATGGGCTCCCTCTCATCAGTTC
TNF-α RV	TCTGCTTGGTGGTTTGCTACGAC
GAPDH FW	CTACATGGCCTCCAAGGAGTAAG
GAPDH RV	CCTCCTCTTCTTCGTCTATGGC

### 3.6 The effect of SMN and SM on changes in IL-2 and TNF-α mRNA expression

**Fig. 6:**
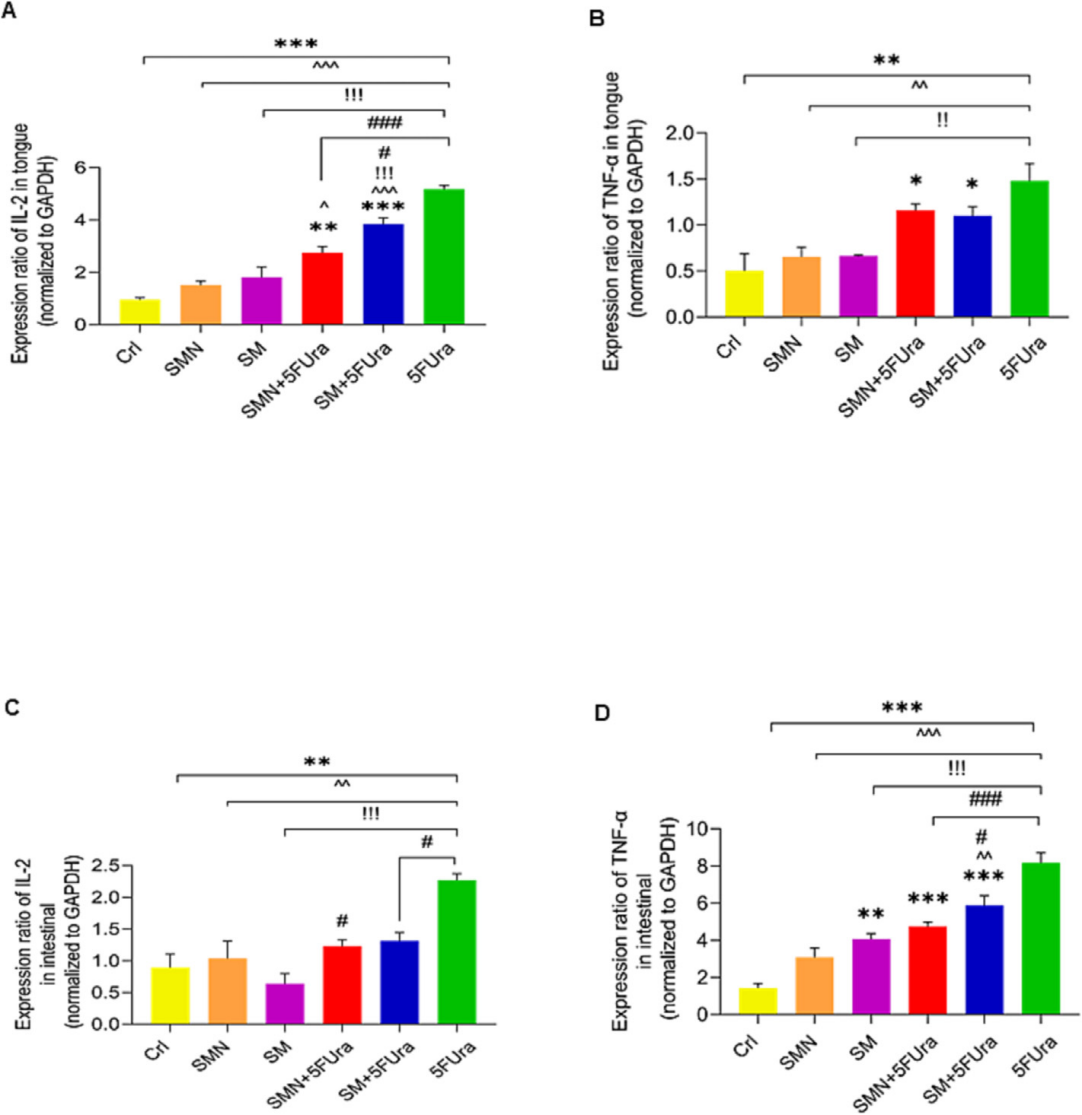
The effect of treatment with SM and SMN on IL-2 and TNF-α gene expression. *P-value of less than 0.05, ^**^P-value of less than 0.01, and ^***^P-value of less than 0.001: significant in comparison to the Crl group. ^^^P-value of less than 0.05, ^^^^P-value of less than 0.01, and ^^^^^P-value of less than 0.001: significant in comparison to the SMN group.^!!^P-value of less than 0.01,^!!!^P-value of less than 0.001: significant in comparison to the SM group. ^#^P-value of less than 0.05, ^###^P-value of less than 0.001: significant in comparison to the 5FUra group. Results are represented as mean ± SEM (n = 5). **A:** IL-2 expression in the tongue. **B:** TNF-α expression in the tongue. **C:** IL-2 expression in the intestine. **D:** TNF-α expression in the intestine.

The mRNA expression of IL-2, and TNF-α in the tongue tissue as well as IL-2 and TNF-α mRNA expression in the intestinal tissue of the 5FUra group were substantially greater than those in the SMN, SM, and Crl groups (Fig. 6).

Regarding the IL-2 gene, an enhancement in IL-2 expression was indicated in the tongue tissue of the SM + 5FUra group in comparison to the SMN, SM, and Crl rats. There was also a markedly higher level of IL-2 in the tongue tissue in the SMN + 5FUra group than that of the SMN and Crl groups. Although, a remarkable decrement in IL-2 level was observed in the tongue tissue in the SMN + 5FUra and SM + 5FUra groups in comparison to the 5FUra group. Moreover, the gene expression of IL-2 was reduced considerably in the intestinal tissue in the SMN + 5FUra and SM + 5FUra groups in comparison with the 5FUra group (Fig. 6. A, C).

Regarding TNF-α in the tongue tissue, the results demonstrated that the TNF-α expression was markedly greater in the SMN + 5FUra and SM + 5FUra groups than in the Crl group. Furthermore, the TNF-α expression was substantially higher in the SM + 5FUra group than in the Crl and SMN groups. In addition, the expression of TNF-α was raised in the SMN + 5FUra and SM groups in comparison to the Crl group (Fig. 6. B, D). A substantial diminution in TNF-α mRNA expression was found in the SMN + 5FUra and SM + 5FUra groups in comparison to the 5FUra treated group (Fig. 6. B, D).

## 4 Discussion

Historically, mucositis was considered to be the result of epithelial damage. It has been assumed that chemotherapy or radiation targeted the quickly proliferating cells of the basal epithelium, destroying the tissue's ability to renew itself. The ulceration, atrophy, and thinning of the mucosal epithelium have been correlated with mucositis and are considered a result of these events. However, the challenge of improving mucosal protection and minimizing the risk of tumor protection will require to be solved.

Natural components isolated from plants have almost been discovered to have efficacious preventive and protective impacts against such pathological states. Silymarin (SM) is a mixture of flavolignans (i.e. silybin, silydianin and silychristin) extracted from *milk thistle Silybum marianum* seeds. Among the flavolignans, silybin or silybinin is the most abundant biologically active compound of silymarin (Zaulet et al., 2017). Various human and animal studies have indicated that SM and its active agent, silybin, act as an antioxidant, a free radical scavenger, and a lipid peroxidation inhibitor by raising superoxide dismutase production and glutathione activity, as well as downregulating inflammatory factors like TNF-α and NF-kB (Altindağ, 2022, Karimi et al., 2021).

However clinical trials have confirmed that silymarin is safe at high doses (>1500 mg/day) in humans, it suffers limiting factors including low solubility in water (less than50 µg/ml), low bioavailability and poor intestinal absorption. To enhance its bioavailability and provide an extended silymarin release at the absorption site, using nanotechnological strategies seems to be a promising way to augment the therapeutic strategy and enhance constant release of the active herbal extract. The study purpose is to evaluate one of the nanostructured systems available as delivery strategies to improve the bioavailability and absorption of silymari and decrease local (mucosal) and systemic levels, respectively (Guo et al., 2003, Rachmawati et al., 2015).

Several researchers concentrated their studies on the design of preconcentrated organic liquid phases loaded with weakly water soluble drugs, capable of creating the emulsions as a liquid self-emulsifying drug delivery system (SEDDS). It means drug must remain partitioned within the oil-in-water (O/W) droplets after dilution with the aqueous medium in the gastrointestinal tract. Otherwise, the drug could undergo an undesirable precipitation leading to poor bioavailability in vivo. One of these options for SM would be polyoxyl 40 caster oil which was selected as a surfactant for its good emulsion-forming capability and smaller droplet size of the optimized SM loaded emulsion (Di Costanzo & Angelico, 2019).

Another optimized SEDDS is using GMO as the oil phase and SMN, with a surfactant mixture of Tween 20 and PEG-X (X = 40, 50) Hydrogenated Castor Oil as cosurfactant (Woo et al., 2007). Also consistent with our study, in a GMO- based formulation, SM was encapsulated in nano-liquid crystals to enhance payload, biocompatibility and biodegradability in brain using GMO, an amphiphilic self-assembling biocompatible lipid employed to encapsulate lipophilic and hydrophilic drugs. This lipid can help to sustain structural cohesion of nanoparticles and nanoemulsions (Luo et al., 2015).

After producing the suitable nanostructure that we needed, first step to determine how inflammatory components and the above- mentioned efficacious preventive ingredients against them would be evaluating macroscopic analysis of oral injury. In our study, macroscopic analysis of the mouth indicated healthy and normal tissue of the tongue and mouth in the Crl, SMN, and SM groups between days 5 and 9. In a study, statistical significance showed a lower value of oral mucositis available using SM, and a lower mean time for oral mucositis healing in SM treated group compared to placebo and chemotherapy-treated groups. Pain relief and ulcer healing have been reported in 100 % of the SM-treated group (Altaei, 2012).

In one of our studies, 5FUra markedly reduced body weight in rats in comparison to the Crls (Safarpour et al.). Besides, based on prior studies, rats that were treated with 5FUra had a substantially lower food intake and body weight owing to the injury to internal organs including the intestine or liver (El-Sayyad et al., 2009) and a reduction in anaerobic gut bacteria (Von Bültzingslöwen, Adlerberth, Wold, Dahlen, & Jontell, 2003). In this study, the comparison of body weight results between the days 1, 5, 9, and 13 demonstrated that 5FUra inhibited weight gain compared with the SMN, SM, and Crl groups during the study. Although SMN and SM inhibited the weight loss in the 5FUra group to some degree. The results have demonstrated that 5FUra hampered gaining weight compared with the SMN, SM, and Crl groups during the study, which illustrates growth retardation.

Another side effect of treating with 5FUra would be inducing oxidative stress and lipid peroxidation, as a result of an interaction between unsaturated fatty acids normally in membrane lipids and free radicals of various origins. The genuine outcome of these events translates into the cumulation of a range of MDA and toxic lipid peroxides. The tissue MDA is often considered a valid lipid peroxidation marker. Oxidative stress is contributed to different kinds of cell death like a certain inducer of apoptosis (Patel, Joseph, Corcoran, & Ray, 2010). In our study, the MDA level was considerably higher in the 5FUra group than that in the SMN, SM, and Crl groups. The biological activities of SM are ascribed to its antioxidant capacity and downstream biological impacts (Velussi, Cernigoi, Dapas, Caffau, & Zilli, 1997). In our study, the levels of MDA in the SM + 5FUra group had a reduction, but this decrease only was considerable in the SMN + 5FUra group compared to the 5FUra group.

Moreover, administrating 5FUra markedly decreased TAC values compared to the SMN, SM, and Crl groups. We showed a substantial reduction in TAC values in the SM + 5FUra group in comparison to the SM group. Besides, in the case of SMN + 5FUra and SM + 5FUra groups, a rise in the TAC level compared with the rats treated with 5FUra was statistically remarkable. Similarly, in Rao and Viswanath’s study, SM (100, 250, and 500 mg.kg^−1^ for one week by gavage) diminished oxidative stress by affecting the antioxidant enzymes including SOD, CAT, GST, and GSH (Rao & Viswanath, 2007).

5FUra can cause histopathological changes in tongue, esophageal, and intestinal tissues. In a previous study, normal tongue tissue was observed in the Crl rats, while tongue histology had considerably degraded in the 5FUra-treated rats (Tefas et al., 2020). In another study, hemorrhage, and hyalinization, in addition to inflammatory infiltrates, were also observed in the tongue of the 5FUra group (Lotfi et al., 2021). Likewise, results obtained from the H & E staining in our study indicated a typical cell morphology in the Crl, SMN, and SM groups without any hyperemia and necrosis. However, the 5FUra group, receiving no protection, illustrated high levels of tissue remarkable histopathological abnormalities and intoxication, including hyperemia, hyaline, and inflammatory cell infiltration in the tongue, esophagus, and intestinal tissues. In the study of Soares et al., 5FUra induced a considerable villus shortening, a raise in intestinal crypt depth and myeloperoxidase activity, and a reduction in GSH concentration and villus-to-crypt ratio (Soares et al., 2008). Besides, we observed mild hyperemia in the tongue tissue in the groups treated with SMN + 5FUra and SM + 5FUra. Furthermore, some degree of hyaline in the tongue tissue was indicated in the SM + 5FUra group. Meanwhile, rats in the SMN + 5FUra and SM + 5FUra groups, particularly in the SMN + 5FUra group, demonstrated less tissue damage than in the 5FUra group, illustrating attenuation of tongue tissue damage and abnormalities in esophageal, and intestinal tissues by SM and especially SMN.

The pathophysiology of mucositis begins with DNA damage by producing ROS. Afterward, raised proinflammatory cytokines like TNF-α, IL-6, and IL-2 induced by NF-kB signaling activation result in tissue injury. As the injury advances, bacterial colonization caused by gram-negative/positive and anaerobic organisms, as well as various components of the bacterial cell wall can stimulate macrophage activation in tissue, which in turn can produce more proinflammatory cytokines (Atawia et al., 2014, Nazemian et al., 2010).

We observed that the mRNA expression of IL-2 and TNF-α in the tongue tissue as well as in the intestinal tissue of the 5FUra group were considerably higher than those in the SMN, SM, and Crl groups. An improvement in IL-2 gene expression was indicated in the tongue tissue in the SM + 5FUra group compared to the SMN, SM, and Crl rats. There was also a substantially higher level of IL-2 in the tongue tissue in the SMN + 5FUra group than that in the SMN and Crl groups. However, a remarkable reduction in IL-2 level was observed in the tongue tissue in the SM + 5FUra and more significantly in SMN + 5FUra groups in comparison to the 5FUra group.

Moreover, the gene expression of IL-2 was diminished considerably in the intestinal tissue in the SMN + 5FUra and SM + 5FUra groups in comparison with the 5FUra group. TNF-α expression was significantly higher in the SMN + 5FUra and SM + 5FUra groups than in the Crl group. Furthermore, TNF-α mRNA expression was substantially higher in the SM + 5FUra group than in the Crl and SMN groups. In addition, the expression of TNF-α was raised particularly in the SMN + 5FUra and SM groups in comparison to the Crl group.

However, suppressing TNF-α production appears to be well associated with efficacious mucositis modulation. On the other hand, SM can block or interrupt the process of mucositis at numerous targets protecting the mucosa and promoting healing of chemotherapy-induced mucositis by decreasing the occurrence, hampering the oxidative stress and anti-inflammatory activities (Altaei, 2012). Likewise, a substantial diminution in TNF-α expression was found in the SMN + 5FUra and SM + 5FUra groups in comparison to the 5FUra treated group.

## 5 Conclusion

Here, we demonstrated that treating with SM, and particularly SMN, may have multiple therapeutic impacts on gastrointestinal protection. We particularly revealed a substantial protective impact of SM and SMN on the oral, esophageal, and intestinal tissues through evaluating histological and biochemical alterations. SM and SMN also prevented weight loss induced by 5FUra. In addition, an improvement in IL-2 and TNF-α mRNA expression was found in the intestine and tongue following SM and SMN treatment. These findings may be beneficial to better investigate the advantages of SM and its nano form, as a novel therapeutic approach for preserving gastrointestinal tissues against chemotherapeutic agent toxicity.

## Funding

This investigation has been financially supported by the research affairs division of the Babol University of Medical Sciences. Grant number: 724132865.

## Declaration of Competing Interest

The authors declare that they have no known competing financial interests or personal relationships that could have appeared to influence the work reported in this paper.

## Data Availability

Data that used to support the study are available from the corresponding author upon request.
